# Optimized Extraction of Insect Genomic DNA for Long-Read Sequencing

**DOI:** 10.3390/mps2040089

**Published:** 2019-11-23

**Authors:** Brenda Oppert, Samantha Stoss, Alaysha Monk, Timothy Smith

**Affiliations:** 1 USDA Agricultural Research Service Center for Grain and Animal Health Research, 1515 College Ave., Manhattan, KS 66502, USA; samantha.stoss@ars.usda.gov (S.S.); alaysha.monk@ars.usda.gov (A.M.); 2 USDA Agricultural Research Service U.S. Meat Animal Research Center, Clay Center, NE 67432, USA; tim.smith@ars.usda.gov

**Keywords:** genomic DNA, long-read sequencing, Oxford Nanopore, PacBio, red flour beetle, *Tribolium castaneum*

## Abstract

Long-read sequencing technologies continue to increase the length of reads, and at present can average read lengths of >20 kb up to 60–80 kb. Now the challenge is to extract genomic DNA of sufficient fragment size and quality to support longer read lengths. We developed a successful method to consistently obtain high-quality long genomic DNA from insects. The optimal developmental stage of insects for genomic DNA extraction was determined to be the pupal stage, eliminating DNA from ingested food and reducing contamination by chitinous material that can interfere with extraction. Improved results were obtained by a modified procedure of a commercial genomic DNA extraction kit. Initially, soft pupal tissue of the red flour beetle, *Tribolium castaneum*, was disrupted in the kit lysis buffer using Teflon micropestles. Modifications to the kit protocol also included gentle mixing by inversion of the tube, instead of harsh vortexing steps, and using wide-bore pipette tips in transferring fractions containing genomic DNA. Data from one sample were provided as an example of successful downstream library production and sequencing. While the technique has been optimized for insects, extractions from tissues of other organisms using these modified procedures also may improve long-read sequencing results.

## 1. Introduction

Long-read sequencing is increasingly being used for a number of biological applications, especially genome assembly. De novo assembly of complex genomes, especially those with highly repetitive sequences that are characteristic of many insect genomes, is improved by the incorporation of long-read (>10 kb) sequences. Protocols that can isolate long segments of genomic DNA while minimizing damage, such as nicks to the DNA strand, are needed to improve long-read sequencing data.

Historically, the first report of extraction of DNA was a referred to as “nuclein” from the cell nucleus and described by Friedrich Miescher [1]. Since that report, there have been many permutations of a basic isolation protocol that begins with the lysis of the cell using surfactants or detergents, removal of protein by a general protease, and removal of RNA by RNase. Removal of protein, RNA, and cell membrane lipids is achieved by salt precipitation and centrifugation. DNA also may be separated from the above contaminants by the addition of alcohol (ethanol or isopropanol), phenol/chloroform, and/or binding to a solid phase such as silica and elution by altering the pH and salt concentration.

A method for the isolation of eukaryotic high-molecular-weight DNA was proposed by Blin and Stafford [2], involving homogenization of tissues in liquid nitrogen in a Waring Blender, and achieving nick-free DNA of 200 × 10^6^ Da (about 300,000 bp). Another method for low cost and rapid genome DNA extraction from plant materials was demonstrated recently [3]. However, the method also used an initial process of grinding tissues in liquid nitrogen, and we have found that this process is not always applicable for insects due to the loss of limited material and damage to genomic DNA.

A number of commercial kits have been developed for rapid and efficient isolation of genomic DNA. For insects, Chen et al. [4] evaluated five different methods for time, efficacy, and cost in the isolation of genomic DNA from the western corn rootworm, a major pest of corn. The methods included the use of SDS or CTAB, or the kits DNAzol (Molecular Research Center, Inc., Cincinnati, OH, USA), Puregene (Gentra Systems, Minneapolis, MN, USA), and DNeasy (Qiagen, Hilden, Germany). While all five methods yielded sufficient amounts of genomic DNA for the intended molecular applications, SDS and CTAB extractions yield larger quantities and exhibit less degradation. All kits had the advantage of not generating hazardous waste of phenol and chloroform, but the DNeasy kit offered the shortest extraction time, and the Puregene kit had lowest contamination of protein. These researchers also found that using up to 8× volumes of ethanol and 4 °C enhanced the amount of DNA extracted, although the increased volume of ethanol increased the cost of extraction, and lower temperatures tended to make the DNA solution more viscous with the potential to clog columns.

We evaluated a number of commercial kits for improvements to genomic DNA extractions from stored product insects for long-read sequencing (data not shown). We found one kit superior in the reproducible extraction of high-quality long genomic DNA, and we demonstrate herein our modified procedure with a common stored product pest, *Tribolium castaneum* (the red flour beetle).

## 2. Experimental Design

Genomic DNA extraction. Among the commercially available kits we evaluated, we found the E.Z.N.A. Insect DNA Kit (Omega BioTek, Norcross, GA, USA) provided the most consistent and superior extraction of genomic DNA from insects. However, the protocol provided with the kit was modified to provide higher quality and longer genomic DNA, as explained in Section 3.

We did not follow the suggested protocol of pulverizing in liquid nitrogen, as we found that we lost sample quantity and quality (data not shown). Instead, 10 male, female, or mixed sex *T. castaneum* pupae (approximately 30 mg) were used as starting material and were ground in the kit lysis buffer. Protein was degraded with proteinase K; DNA was extracted with 24:1 chloroform:isoamyl alcohol and digested with RNAse to remove RNA. Genomic DNA was isolated and purified and a 1 μL aliquot of the genomic DNA samples from *T. castaneum* pupae was analyzed for quality and quantity by a digital nanophotometer and TapeStation.

Samples were transported on ice to the USDA ARS U.S. Meat Animal Research Center in Clay Center, NE for library construction and sequencing on the PacBio Sequel I. The library was prepared with a SMRTbell Template Prep Kit 1.0-SPv3 as recommended by manufacturer, using a 15 kb lower bound cutoff for insert size selection on the BluePippin.

### 2.1. Materials

- Insects. Established colonies of *T. castaneum* are continuously reared at the Center for Grain and Animal Health Research (CGAHR), Manhattan, KS, with the feeding stages (larvae and adults) maintained on 95% wheat flour and 5% brewer’s yeast, 28 °C, 75% relative humidity, complete darkness. The colony used for both genomic DNA extraction and genome sequencing was GA-2, the same colony used in the original genome sequencing project [5]. We extracted genomic DNA from all stages of our stored product insects and determined that the pupal stage is optimal for the extraction of high-quality long genomic DNA because of the lack of food contamination and chitinous material that may clog columns (data not shown). Therefore, early stage pupae (approximately 3 mg) were used for genomic DNA extractions.
- E.Z.N.A. Insect DNA Kit (Omega BioTek, Norcross, GA, USA; Cat. No.: D0926-02).
- Sterile blue pellet pestle (Kimble Chase, obtained from Labsource, Northlake, IL, USA; Cat. No.: 749520-0000).
- 24:1 chloroform:isoamyl alcohol (Acros Organics, obtained from ThermoFisher Scientific, Waltham, MA, USA; Cat. No.: AC327155000).
- 200 and 1000 µL wide-bore micropipette tips (ART^TM^, acquire through ThermoFisher Scientific).
- 100% ethanol 200 proof molecular biology grade (Decon Laboratories, King of Prussia, PA, USA; Cat. No.: 3916).
- SMRTbell Template Prep Kit (Pacific Biosciences, Menlo Park, CA, USA; version 1.0-SPv3).

### 2.2. Equipment

- Shaking incubator (Thermomixer Model 5436, Eppendorf, Hauppauge, NY, USA).
- Tabletop centrifuge (Sorvall Legend MicroCL 21R, ThermoFisher Scientific).
- Digital nanophotometer Model NP80 (Implen, Westlake Village, CA, USA).
- Tapestation Model 2200 (Agilent, Santa Clara, CA, USA).
- PacBio Sequel I (Pacific Biosciences).
- BluePippin (Sage Science, Beverly, MA, USA).

### 2.3. Costs

Samples can be extracted for $2.29 to $3.60 per sample, depending on the kit size. Additional costs not included include the consumables (pestles and tips) and 24:1 chloroform:isoamyl alcohol ($309.50 for a 500 mL bottle).

## 3. Procedure

### 3.1. Disrupt Tissue (5 min)

Combine tissue (less than 25 mg) with 350 μL of CTL buffer from the E.Z.N.A. Insect DNA Kit in a 1.5 mL sterile microcentrifuge tube and grind by hand for approximately 2 min with a sterile blue pellet pestle.

### 3.2. Digest Protein (1–12 h)

Add 25 μL of the proteinase K solution from the kit to the tissue ground in CTL buffer, mix gently by carefully inverting the tube 10 times, and incubate at 60 °C for 1 h in a shaking incubator set at low speed. Incubation at room temperature overnight on an orbital mixer can increase yield.

### 3.3. Extract DNA (10 min)

Add an equal volume (350 μL) of 24:1 chloroform:isoamyl alcohol to the sample. Instead of the recommended vortexing, invert gently 20 times to mix thoroughly. Centrifuge at 10,000× *g* for 2 min at room temperature in a tabletop centrifuge. Transfer the upper aqueous layer carefully, avoiding any milky precipitate that may form on the interface, 100 µL at a time, to a clean 1.5 mL microcentrifuge tube using a 200 µL wide-bore pipette tip (in our experiment, approximately 250 µL in total was transferred).

### 3.4. RNAse Digestion (15 min)

Add one volume of BL buffer and 2 µL RNase A from the kit. Instead of vortexing, gently invert the sample 20 times, and incubate at 70 °C for 10 min.

### 3.5. Extract DNA (5 min)

Add one volume (in our case, 500 µL) of 100% ethanol 200 proof molecular biology grade to the sample. Instead of vortexing, gently invert the sample 20 times. At this point, you may be able to visualize the floating strands of translucent DNA.

### 3.6. Purify DNA (30 min)

Add 500 μL of the sample with a 1 mL wide-bore pipette tip to the HiBand DNA Mini Column inserted into a 2 mL collection tube (both provided in the kit), and centrifuge at 15,000× *g* for 1 min. Discard the filtrate, and repeat with the remainder of the sample (in our case, 500 μL) and the same HiBand column.

After all of the sample is transferred, transfer the column to a sterile 2.0 mL collection tube also provided in the kit, and add 500 μL HBC buffer from the kit (be sure that the HBC buffer has been diluted with isopropanol according to kit instructions). Centrifuge at 15,000× *g* for 1 min. Discard the filtrate and add 700 μL DNA Wash buffer from the kit (diluted according to kit instructions with 100% ethanol). Centrifuge at 15,000× *g* for 1 min and repeat the wash step once.

After the washing steps, spin the column for an additional 2 min at 15,000× *g* to dry the column matrix. Transfer the column to a sterile 1.5 mL microcentrifuge tube, carefully add 50 μL of the kit Elution buffer that is preheated to 70 °C to the center of the column membrane, and incubate at room temperature for 5 min. Elute the genomic DNA from the column by spinning at 15,000× *g* for 1 min; repeat the elution process once. Assess the genomic DNA for quality and quantity and store at room temperature for immediate use. If necessary, sample can be frozen at −80 °C, but the length and quality of the genomic DNA may be affected.

## 4. Results

**Figure 1:**
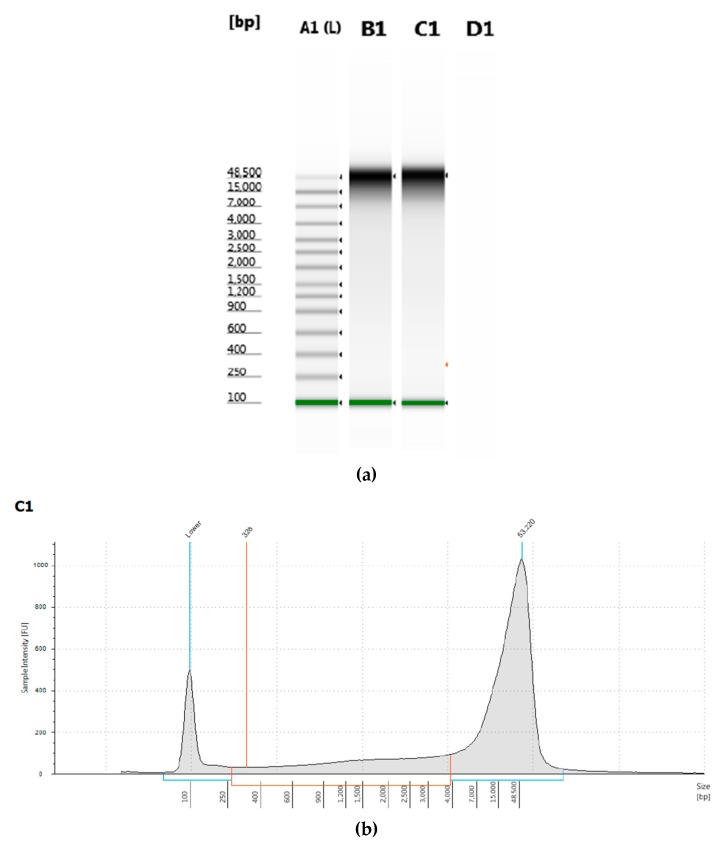
Analysis of genomic DNA. (**a**) Electrophoretic gel of genomic DNA extracted from male *T. castaneum*; (**b**) trace from sample C1, which was used for library construction and sequencing on the Sequel.

Genomic DNA from *T. castaneum* pupae was successfully obtained using a modified procedure of a commercial kit. The DNA had optimal A_260_/A_280_ and A_260_/A_230_ ratios recommended for long-read sequencing (Table 1). The concentration of genomic DNA obtained from female *T. castaneum* pupae was approximately twice that obtained from male pupae. The genomic DNA extracted from *T. castaneum* pupae was measured by electrophoresis and had a peak of greater than 50 kb length (Figure 1).

**Figure 2:**
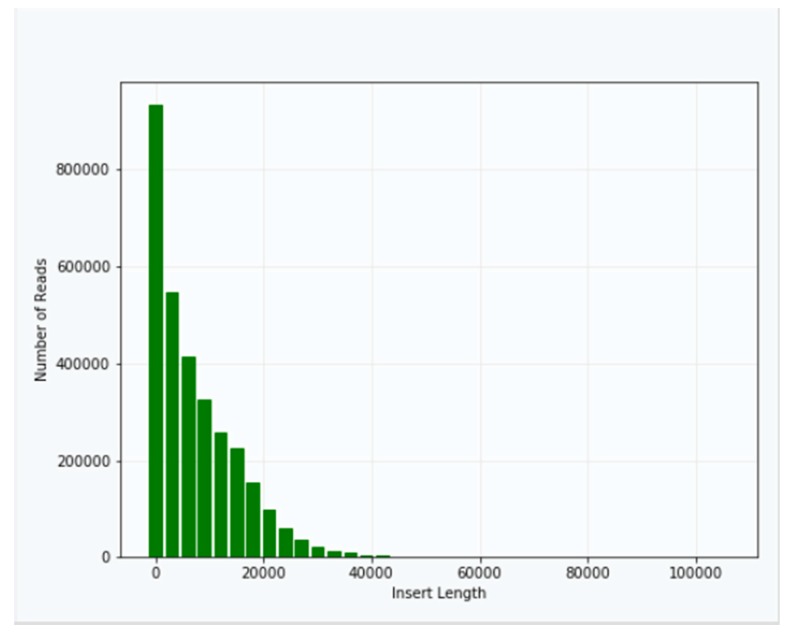
Distribution of insert lengths in the library from *T. castaneum* genomic DNA.

**Figure 3:**
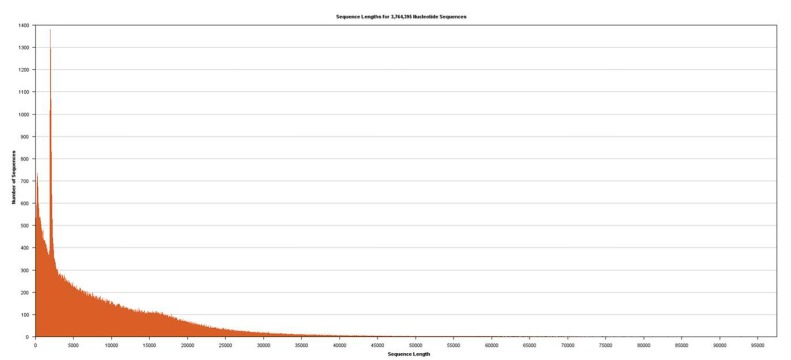
Distribution of lengths from sequences obtained from *T. castaneum* genomic DNA.

Approximately 40 μg of genomic DNA extracted from sample B1 was used for library production and sequencing. The mean insert length of the library was 8731, with insert N_50_ = 14,750 (Figure 2). Sequencing was performed using Sequel v2.1 chemistry, obtaining a total of 3,764,395 subreads from 30.0 Gb, with an average length of 7970 (Figure 3).

## 5. Discussion

We present a rapid and reproducible method to obtain long genomic DNA with high quality from insect pupae. After extractions from different life stages of insects, we found that pupae provided the best quality sequences devoid of food, and extractions using columns were less likely to clog due to chitinous material.

The protocol was easy to execute and took slightly over two hours to complete without the overnight incubation period. The main changes that we made to the recommended kit protocol were:

- Homogenizing tissue in CTL Buffer with a pestle instead of grinding in liquid nitrogen;
- Always using wide-bore pipettes in moving any samples containing genomic DNA;
- Never vortexing, always mixing by gentle inversion.

The kit included a number of methods to increase DNA yield, such as incubation of elution buffer on the column for 5 min prior to elution, and repeating the elution step, both of which were used in our procedure. Also recommended was to increase the elution volume beyond 100 μL, which we did not choose so as not to dilute the sample.

There were several considerations in modifying the protocol to increase yield and improve quality of the genomic DNA. It was critical to grind samples sufficiently to maximize yield of genomic DNA, but we found that grinding in liquid nitrogen and transfer resulted in lower yields, and sometimes lower quality. Manual grinding with a micropestle in a lysis buffer for several minutes yielded higher amounts and quality genomic DNA. While the kit suggested that incubation with proteinase K up to four hours may be sufficient to remove contaminating protein, we found that overnight incubation improved the absorbance ratios in the quality assessment; however, it was necessary to avoid vigorous shaking during the incubation period. The use of wide-bore pipette tips was absolutely critical in transferring genomic DNA to avoid shearing; if wide-bore tips are not available, use a sterile razor blade to slice and widen the opening in the end of the tip. Another important step to avoid shearing of DNA was to always invert tubes gently, and never vortex. As recommended in the kit protocol, use of newly aliquoted ethanol was necessary for adequate DNA precipitation. These recommendations provided improvements to the successful purification of genomic DNA from *T. castaneum* for long-read sequencing, and also have been used successfully with other insect species.

## Figures and Tables

**Table 1:** Representative absorbance ratios and concentration from genomic DNA extracted from male and female *T. castaneum* (samples were diluted 1:20).

Sample	A260/A280	A260/A230	Concentration (ng/μL)
Male	1.958	2.043	23.5
Female	2.040	2.000	51.0
